# Monitoring the past and choosing the future: the prefrontal cortical influences on voluntary action

**DOI:** 10.1038/s41598-018-25127-y

**Published:** 2018-05-08

**Authors:** H. N. Phillips, T. E. Cope, L. E. Hughes, J. Zhang, J. B. Rowe

**Affiliations:** 1 Department of Clinical Neurosciences, University of Cambridge, Cambridge, CB2 0SZ UK; 2 Medical Research Council, Cognition and Brain Sciences Unit, Cambridge, CB2 7EF UK; 3 School of Psychology, University of Cardiff, Cardiff, CF2 2AT UK; 4 Behavioural and Clinical Neuroscience Institute, University of Cambridge, Cambridge, UK

## Abstract

Choosing between equivalent response options requires the resolution of ambiguity. One could facilitate such decisions by monitoring previous actions and implementing transient or arbitrary rules to differentiate response options. This would reduce the entropy of chosen actions. We examined voluntary action decisions during magnetoencephalography, identifying the spatiotemporal correlates of stimulus- and choice-entropy. Negative correlations between frontotemporal activity and entropy of past trials were observed *after* participants’ responses, reflecting sequential monitoring of recent events. In contrast, choice entropy correlated negatively with prefrontal activity, *before* and *after* participants’ response, consistent with transient activation of latent response-sets ahead of a decision and updating the monitor of recent decisions after responding. Individual differences in current choices were related to the strength of the prefrontal signals that reflect monitoring of the statistical regularities in previous events. Together, these results explain individual expressions of voluntary action, through differential engagement of prefrontal areas to guide sequential decisions.

## Introduction

The brain is adept at identifying and representing regularities within a dynamic sensory environment, such as the identification of rhythms in auditory streams^1,2^, recurrent visual features embedded in complex objects^3^, and the transitional relationships between elements in artificial grammars^4^. Implicit learning of the statistics of event regularity is evident from early in development^5^. These expectations adaptively influence behavior, and facilitate preferential responses to new events^6^.

Statistical regularities span different timescales, which map onto a rostral-caudal gradient of neural representations^7^. While the analysis of shorter sequences relies on the basal ganglia^8,9^, regularities from temporally extended sequences (tens of seconds) have been associated with the prefrontal cortex^10,11^. The neural response to regularities is established across multiple sensory modalities^12,13^, whether in fixed event blocks^14,15^, over all previous events^16,17^ or during varying time windows^11^.

Sequential voluntary actions also contain statistical regularities. Where action decisions cannot be explained by objective differences in outcome or reward, individual differences in the degree of regularity provide critical insights into the mechanisms of volition^18^. Volition is integral to normal human behavior, and many neurological disorders are characterized by changes in volition, with corresponding differences in regularity, entropy or stereotypy of behaviors^19^. This study therefore lies in the broader context of willed action and volitional decision making. Voluntary actions encompass everyday decisions that are not by reflex or forced by some external stimulus or specified rule (over and above the willingness to adhere to such instructions)^20^. They are sometimes considered internally-driven decisions or consciously attended to^21,22^ or associated with a sense of agency when making choices between possible options^23,24^. However, terms such as “free-will” or “free selection” of action are poorly operationalized, and open to highly variable interpretation: there are often implied or actual constraints on the range of actions from which to choose. Instead, we propose analysing such tasks in terms of decision-making and choice. Cortical regions consistently associated with action selection include parietal^25,26^ premotor^27,28^ and prefrontal areas^29,30^. Conversely, abnormal statistical dependencies in the form of perseveration and stereotypies are often associated with dysfunction of the prefrontal cortex and its striatal connections^31^, including Parkinson’s disease and progressive supranuclear palsy^32^, Tourette syndrome^33^ and frontotemporal dementia^34^.

Based on an fMRI study of voluntary action selection, we recently proposed two mechanisms by which the prefrontal cortex introduces regularity to sequential voluntary behaviors^11^. Firstly, by monitoring serial actions, it introduces a bias towards selection of previously under-represented choices^35^. Secondly, it facilitates the implementation of transient and arbitrary response rules. Such rules are not essential for voluntary action, but may serve to reduce the effort required to resolve ambiguity where the selection between action alternatives is not facilitated by differential rewards^36–39^. A simple rule might be the inhibition of repetition of sequential choices^18^ analogous to inhibition of return demonstrated in attention and saccades^40,41^. For example, Zhang *et al*.^18^ demonstrated that prefrontal cortical activation brakes the activation of premotor representation of recent actions, leading to regularising of behavior.

Neuropsychological and fMRI studies are not able to determine whether prefrontal cortical activity related to selection regularity occurs before the selection of action or afterwards. Regularisation activity before the response suggests a constraint on the current choice, for example by a transient rule that reduces effort by minimising uncertainty^42^. In contrast, regularisation related activity after the response suggests the monitoring of behavior, or the updating of a heuristic response-set.

We therefore exploited the temporal resolution of magnetoencephalography (MEG) to investigate how the degree of regularity in past events modulates present evoked responses. Using a task in which participants are instructed to make a specified action or are given a choice of actions to make, we used entropy to measure types of regularity, quantified as the degree of regularity in past trial events (Trial Entropy, TE) and the degree of regularity of participants’ voluntary action decisions (Selection Entropy, SE). Entropy measures do not depend on transition probability, which suits the current experiment given the design that interleaves choice and specified trial types.

Tobia *et al*.^15^ puts forward in their fMRI study that entropy quantifies uncertainty, the inverse of predictive mechanisms used in higher cognitive processes. For example, they interpret positive correlations between past randomness neural activity as an increase of prediction error signals^43^ and negative correlations as regions that monitor predictability of current events given past events^14,15^. We expected to observe neural correlates of TE and SE in temporal and prefrontal regions in replication of a previous fMRI experiment using a similar multi-choice action selection task^11^. Crucially, the high temporal resolution of MEG enabled us to test whether (1) entropy-related neural activity in sequential action selection occurs before or after the action; and (2) whether individual behavioural differences can be explained by monitoring of the preceding regularity in either trial type (TE) or subjects’ action choices (SE).

## Results

### Behavioral results

**Figure 1:**
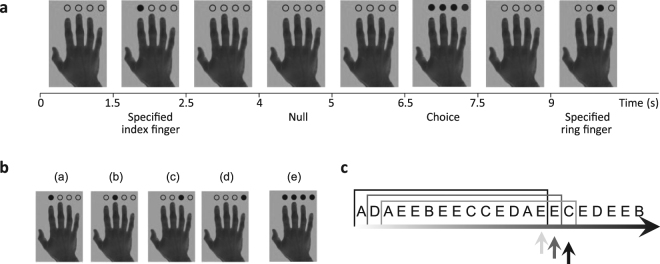
The four-choice action selection experiment design. (**a**) An example trial sequence with specified (one circle filled), choice (four circles filled) and null trials (no circles filled). The image of the hand with unfilled circles remains on screen between trials for 1.5 seconds. Each stimulus trial is presented for 1 second, with 2.5 second stimulus onset asynchrony. (**b**) The experimental stimuli used in the action selection task. Trials (a–d) are the specified trial cues where the participant was cued to press the specified finger. Trial (e) is the choice trial cue where the participant was cued to make an action with a finger of their choice. (**c**) We show an example of a trial window incrementally sliding over trial stimuli (a–e in B) to calculate the entropy of stimuli or actions preceding the current trial. The trial entropy (TE) and selection entropy (SE) values were assigned to the last trial within the window as the arrows show. Figure adapted with permission from^11^.

We recorded MEG data from 18 healthy young participants completing a multi-choice action selection task (Fig. 1). Participants were instructed to press the button for a specific finger in ‘Specified’ trials or were to make a new fresh choice of which button to press for ‘Choice’ trials. On average, participants responded in 99.0 ± 1.2% of action trials and the average total error rate was 3.2% for omission and commission errors. As expected^18^, participants’ mean reaction times for choice trials was slightly longer than for specified trials (584 ms ± 76 ms and 566 ms ± 56 ms respectively; two-tailed paired t-test t(17) = −2.10, p = 0.05). We assessed the effect of finger selection on reaction times using a repeated-measures ANOVA with two factors: finger selection (index to little finger) and task condition (specified or choice). There was no significant main effect of finger (F(1.87, 31.7) = 3.32, p = 0.52), or task condition (F(1, 17) = 4.45, p = 0.50), but there was a significant interaction between finger and task condition (F(2.07, 35.2) = 11.4, p < 0.001) such that, during choice trials, participants selected each finger with the following probabilities: index = 26.8%, middle = 28.7%, ring = 27.5%, little = 17%. Post-hoc tests demonstrated that the middle and little finger actions were significantly different from 25% chance rate (Middle: Z = +2.85, p = 0.004; Little: Z = −3.72, p < 0.001, one-sample Wilcoxon signed-rank test).

During choice trials, participants tended to choose a new action rather than repeat the previous action (repetition rate: 12.2 ± 12.6%; Z = −3.28, p < 0.001, against chance rate of repetition at 25%, one-sample Wilcoxon signed-rank test). This inhibition of repetition was concordant with previous studies, and suggests that a current choice is modulated by the previous response history^18,44^. The repetition rate was not significantly different across fingers (F(1.96, 33.6) = 0.28, p = 0.75) and the probability of finger choices were not different across repeated and non-repeated trials (χ^2^ (17) = 1.67, p = 0.543, Friedman’s test).

### Trial and Selection entropy

**Figure 2:**
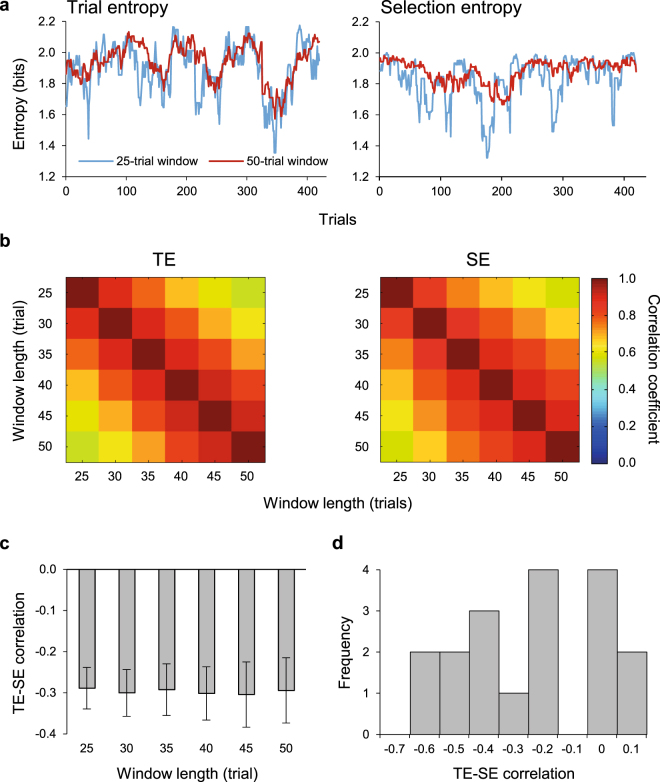
Entropy measures. (**a**) Trial entropy (TE, left) and selection entropy (SE, right) for a single participant. The blue lines are entropy measures using the 25-trial sliding-window and the red lines are the 50-trial entropy measures. (**b**) Correlation of the different sliding windows for TE (left) and SE (right), averaged across all participants. All correlations were significant (p < 0.003, even with conservative Bonferroni correction, noting that the entropy measures are not independent tests between sliding windows of different length). (**c**) The mean Fisher transformed correlations between TE and SE with standard error bars. Each correlation was significant. (**d**) A histogram showing frequency of TE-SE correlation across participants for the 25-trial window. Some individuals displayed a strong negative correlation between TE and SE, while others demonstrated a weaker or no relationship.

To observe the neural representation of regularity monitoring we measured the entropy of past trials events (Trial Entropy, TE) and of past finger choices (Selection entropy, SE) across temporally extended periods, we examined six windows of 25–50 trials, in steps of 5. These window lengths were chosen to obtain meaningful measures of entropy and encompass those used in previous studies of statistical information representations in the brain for temporally extended event sequences^11,16,45^. All participants showed fluctuations over time in their SE values. Their TE values also fluctuated over time because trial conditions were pseudo-randomly intermixed. Figure 2a shows an example single participant’s TE and SE values for the shortest (25) and longest (50) sliding window lengths. The entropy measures for each window were non-independent, i.e. data from the 25-trial window and 30-trial window overlap in all but five trials. Thus, entropy measures were significantly correlated across time (Fig. 2b, Pearson’s r > 0.55 and r > 0.65 for TE and SE respectively, p < 0.003, Bonferroni corrected), where neighboring window lengths had highest coefficients.

We tested whether entropy influenced reaction times using a within-participant Pearson’s correlation between single-trial reaction times and the corresponding trial and selection entropies. We used reaction times here as an indication of participant’s attention across the task, where we would expect slower reaction times with reduced concentration. We observed no significant correlations for any window lengths for either SE or TE (r < ±0.018, p > 0.20 across all participants), suggesting that TE and SE measures were not significantly confounded by trial-to-trial variations in reaction time and therefore attention.

There was a significant negative correlation between TE and SE for each window length (Z < −2.67, p < 0.007, Fig. 2c). There was not a significant main effect of window length on the TE-SE correlations (χ^2^(5) = 2.63, p = 0.76, Friedman’s test). Therefore, although SE was conditional on trial type, the recent specified trials order partially influenced the current trial choice. The strength of this relationship was observed to vary between individuals. For a window length of 25, where the overall group correlation between TE and SE was z = −0.297, single participant correlations ranged from z = 0.021 to z = −0.605. Some individuals therefore displayed a strong negative correlation between TE and SE, while others demonstrated a weaker or no relationship (Fig. 2d). No participants displayed a significant positive correlation. Hartigan’s dip test^46^ over 10000 iterations confirmed that this represented a unimodal distribution suitable for further parametric analysis, rather than a bimodal distribution of strong and weak responders (dip = 0.105, p = 0.079).

### Entropy related MEG responses

Single trial gradiometer sensor MEG data were correlated with TE and SE measures using a first-level statistical parametric mapping (SPM) general linear model. For SE and TE separately, the resulting contrast images were used in a second-level SPM full-factorial model across all participants and all window lengths.

**Figure 3:**
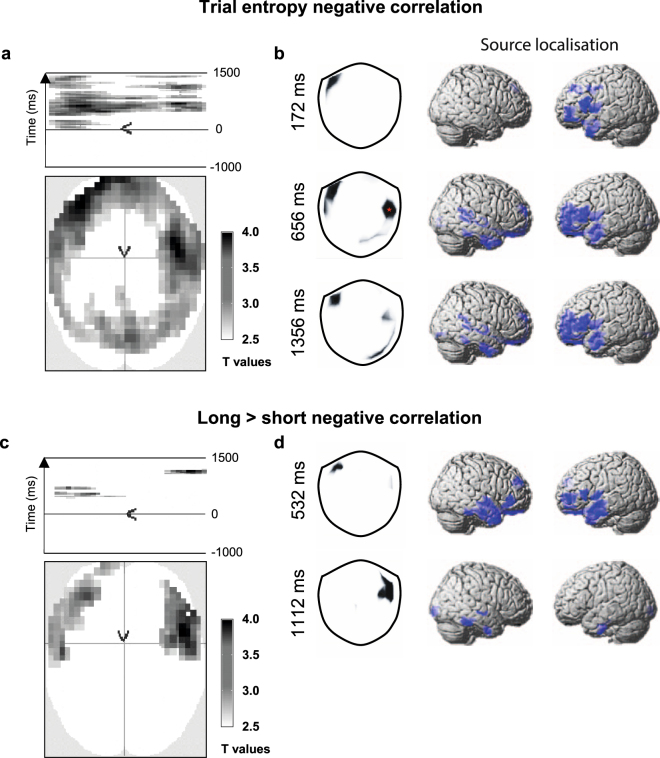
Trial entropy correlations with MEG. (**a**) The significant negative correlations between TE and planar gradiometer data, averaged across all TE windows in sensor-time space (voxel threshold: p < 0.001, t > 3.17 with FWE cluster thresholded at p < 0.05). The scalp plot shows the sensor space clusters collapsed across time and the figure above shows the x plane of sensor space against time. Note that all significant correlations were observed after the response time at t = 0 ms. (**b**) The t-maps (left) show those scalp locations at which negative correlations were above threshold at the peak time point of each cluster. The location of peak overall group response across the whole of scalp-time space is indicated by a red star. We visualised the location of the neural sources (right) within a 20 ms time window around each of these time-points (p < 0.01, t > 2.36). At t = 172 ms, source peaks were observed in the left inferior frontal gyrus, and anterior middle frontal gyrus (Neuromorphometrics atlas). Similar peak locations were observed for t = 656 ms and t = 1356 ms in left anterior middle frontal gyrus, right superior temporal gyrus and left temporal pole. (**c**) Clusters in sensor-time space with significantly greater negative correlations for longer trial windows (thresholding as panel a). (**d**) The t-maps (left) show the scalp location of the significant differences between window lengths at the peak time point for each cluster. For the t = 532 ms peak, the sources were localised to the left anterior middle frontal gyrus and bilateral superior temporal gyrus (thresholding as panel b). For the t = 1112 ms peak, the largest cluster was localised to the right inferior temporal gyrus.

For trial entropy, TE, there were significant negative correlations with the MEG responses (Fig. 3a, p < 0.001 threshold with p < 0.05 FWE cluster correction). Crucially, these correlations began 30 ms *after* the participants’ response and continued until the end of the epoch (1500 ms). For MEG gradiometer sensors, the measurement at the scalp is maximal over the source of neural activity^47^, which gives a fair approximation of the location of cortical sources in sensor space. The Fig. 3b sensor space t-maps show these correlations were observed over the left frontal sensors throughout the post-response period, and additional right frontotemporal sensors later 656–1356 ms period.

To visualise the neural sources of these statistically significant sensor space correlations, we performed minimum norm source reconstruction of the single-trial MEG data around the time of peak effect within the significant sensor space clusters. We then correlated the resulting source space images with the trial entropy measures (Fig. 3b right, p < 0.01). At 172 ms, we observed correlations of trial entropy within the left inferior frontal gyrus (MNI: [−50, 10, 2], Neuromorphometrics Atlas, SPM12), and anterior middle frontal gyrus [−40, 46, 0]. Similar peak locations were observed for both 656 ms and 1356 ms time points in left anterior middle frontal gyrus ([−36, 52, 2] and [−22, 58, 0] respectively), right superior temporal gyrus ([52, −38, 10] and [54, −44, 12]) and left temporal pole ([−48, −2, −26] and [−50, 0, −28]).

We contrasted the short (25) and long (50) window lengths. Figure 3c shows significantly stronger negative correlations in bilateral frontotemporal sensor regions for longer trial windows. The left frontal sensors had negative correlations that peaked after the response at 532 ms, and were localised to the left anterior middle frontal gyrus [−40, 48, 2] and bilateral superior temporal gyrus ([−42, −8, −14] and [56, −10, −10]). The right frontal sensor negative correlations peaked later at 1112 ms, and were localised to the right inferior temporal gyrus [52, −44, −26]. No correlations were greater for short vs. long windows. We observed no significant positive correlations between TE and the MEG response.

**Figure 4:**
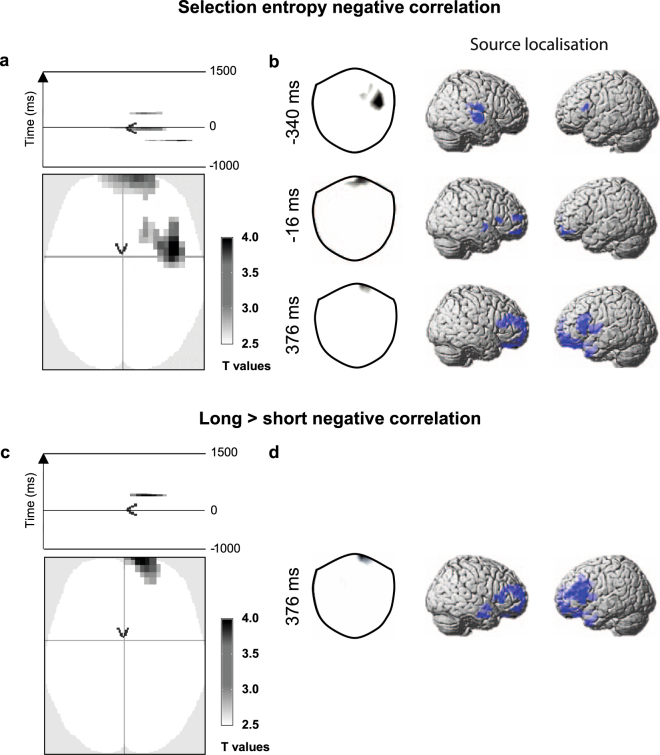
Selection entropy correlations with MEG. (**a**) The significant negative correlations between SE and planar gradiometer data, averaged across all SE windows in sensor-time space (voxel threshold: p < 0.001, t > 3.17 with 50 voxel cluster thresholding^11^). Negative correlations were observed both before and after the response at t = 0 ms. (**b**) The t-maps (left) show the scalp location of the significant correlations at the peak time point of each cluster. We visualised the location of the neural sources (right) within a 20 ms time window around each of these time-points (p < 0.01, t > 2.36). At t = −340 ms, source peaks were observed in the right central operculum and left inferior frontal gyrus. At t = −16 ms, source peaks were observed in right anterior middle frontal gyrus, left bilateral anterior orbital gyrus and right superior temporal gyrus. At t = 376 ms source peaks were observed in the left anterior orbital gyrus, right frontopolar prefrontal cortex, left inferior frontal gyrus and left temporal pole. (**c**) Clusters in sensor-time space with significantly greater negative correlations in the frontal pole for longer trial windows (thresholding as panel a). (**d**) The t-maps (right) show location of the significant differences between window lengths at the peak time point of the significant cluster. This was localised (right) to the left inferior frontal gyrus, left anterior orbital gyrus and right superior frontal gyrus (thresholding as panel b).

For selection entropy, SE, we observed negative correlations both *before* the participants’ response at right and polar frontal sensors, and *after* the response at frontal polar sensors (Fig. 4a and b), but these did not survive FWE cluster correction threshold. However, the use of such stringent whole-brain correction does not reflect our strong *a-priori* expectation for frontal lobe correlations with SE on the basis of previously published fMRI results^11^. In Fig. 4 we therefore present results with the same height threshold but a more lenient 50-pixel cluster defining threshold. We again visualised the sources of these correlations using minimum norm source localisation (Fig. 4b, p < 0.01). At −340 ms, source peaks were observed in the right central operculum [58, −14, 16] and left inferior frontal gyrus [−54, 20, 18]. At t = −16 ms, source peaks were observed in right anterior middle frontal gyrus [22, 50, 14], bilateral anterior orbital gyrus ([−24, 44, −12] and [22, 52, −16]) and right superior temporal gyrus [58, −2, −2]. At t = 376 ms source peaks were observed in the left anterior orbital gyrus [−24, 46, −12], right superior frontal gyrus [12, 62, 20], left inferior frontal gyrus [−38, 14, 24] and left temporal pole [−28, 12, −36].

Given our *a-priori* hypothesis of the presence of negative correlations in the frontal pole from previous fMRI observation^11^, we assessed the effect of window length within a 20mm box ROI in the frontal pole. The post-response signal in the frontal polar region was significant for the long > short contrast (p = 0.009, t = 3.48, FWE peak correction, Fig. 4c). Source localisation (Fig. 4d), suggested that the strongest negative correlations were in the left inferior frontal gyrus [−36, 14, 24], left anterior orbital gyrus [−20, 58, −14] and right superior frontal gyrus [12, 58, 22]. There were no significant differences for the reverse contrast or for positive correlations with MEG.

### Inter individual variability

**Figure 5:**
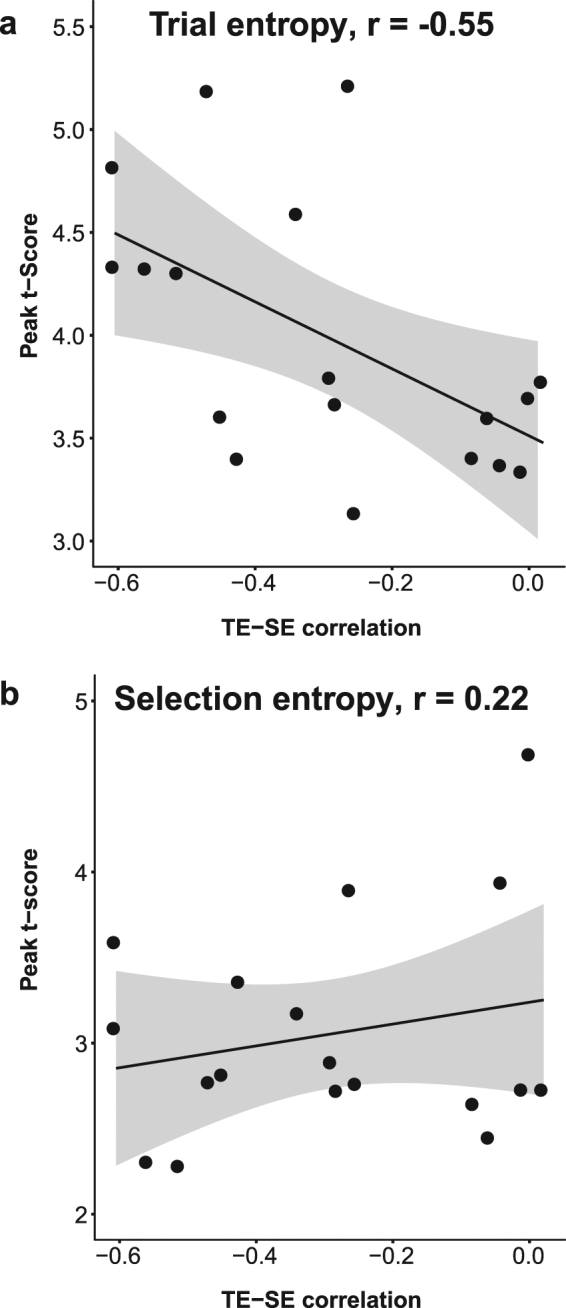
The correlations between MEG peak t-scores for TE (**a**) and SE (**b**) and the TE-SE correlations for individual participants. Best fit linear regression lines and their standard errors are superimposed. There is a significant negative correlation for TE (Pearson’s r = −0.55, n = 18, p = 0.018) but not for SE (Pearson’s r = 0.22, n = 18, p = 0.38).

As expected from previous work, we observed that TE and SE were negatively correlated^11^. The strength of this relationship differed between individuals (Fig. 2d). In a post-hoc exploratory analysis, we explored the neural correlates of this difference. Figure 5 shows the between-participants relationship between the peak t-score of each entropy-related neural response (Figs 3a and 4a) and the correlations between TE and SE for the 25-event window (Fig. 2d). We demonstrated a significant negative correlation for TE (Pearson’s r = −0.55, n = 18, p = 0.018) but not for SE (Pearson’s r = 0.22, n = 18, p = 0.38). We compared these correlations using Meng’s z-test for comparing correlations^48^. The TE and SE correlations were significantly different (z = 2.31, p = 0.011).

Shapiro-Wilk tests demonstrated that the use of parametric statistics to assess correlations between our measures of interest was not rendered inappropriate by deviations from the normal distribution in these variables: (TE peak t-score W = 0.92, n = 18, p = 0.11; TE-SE correlation W = 0.90, n = 18, p = 0.055). However, to ensure the robustness of this finding, the analyses were repeated using non-parametric Spearman’s rank correlations. The results were replicated, with a significant negative correlation again demonstrated for TE (Spearman’s rho = −0.56, n = 18, p = 0.018) but not for SE (Spearman’s rho = 0.06, n = 18, p = 0.80).

Similarly, the results were robust to different choices in how the strength of neural response related to TE was quantified for each individual. We used a binary mask of group significance to restrict the time and location of the single subject analysis to those clusters demonstrated to have population-level relevance for TE and SE respectively. Repeating the analysis with a much more liberal mask, containing any pixel with p < 0.001 correlation at the group level uncorrected for multiple comparisons and therefore resulting in more latitude for individual differences in the scalp location and time of peak response, had very little effect on the negative correlation demonstrated for TE (Pearson’s r = −0.54, n = 18, p = 0.021; Spearman’s rho = −0.54, n = 18, p = 0.023). The t-peak values were determined for each individual separately, not on the group t-maps, on the basis that it could not reasonably be expected that all individuals would display the exact time and scalp location of response. However, to assess the uniformity of response we repeated the analysis with a much more stringent mask restricted to the group peak ([X = 47, Y = 8, time  = 656 ms, F(1,102) = 29.74, p < 0.001] +/− 1cm and +/− 50 ms; marked with a star in Fig. 3b) and took the median individual t-score within this mask as the dependent measure for each individual. This resulted in slightly weaker correlations (Pearson’s r = −0.45, n = 18, p = 0.061; Spearman’s rho = −0.53, n = 18, p = 0.026), but a similar pattern.

Overall, therefore, individual differences in the strength of the relationship between past events and future choices could be accounted for by the strength of neural monitoring of past events (TE) but not by the strength of monitoring of past choices (SE).

## Discussion

When faced with a choice between similar alternative actions, our decisions are constrained by the history of recent experience and choices. These constraints vary over time, leading to slow fluctuations in the regularity of behavioral decisions. This study makes three key contributions. First, we replicate the finding that frontal and temporal neural responses relate to the regularity in the sequence of recent events (trial entropy). But, by exploiting the temporal resolution of MEG, we demonstrated that these correlations occur *after* the response, suggesting the updating of a monitor of recent stimulus events, occurring after the new action is made. Second, we replicate the finding that rostral frontal activity correlated negatively with the entropy of participants’ own chosen actions (selection entropy), but here we demonstrated that the physiological response occurred both *before* and *after* the response. Third, the degree to which individuals manifested a link between the variability of the preceding experimental context and their current behavior was related to the strength of their neural correlates of trial entropy but not selection entropy. We interpret this as evidence that the constraints on current behavioral choices are driven more strongly by the degree of monitoring of recent events than by the instantiation of arbitrary rules.

The prefrontal cortex facilitates optimal interactions with a dynamic environment^10,49,50^. By virtue of its connectivity, this region is well placed to integrate sensory information from multiple domains, defining behavioral goals, maintaining the response sets necessary to achieve them, and predicting the outcomes of action^49,51^. The value of a given course of action may be learned by subjects, either in association with specific stimuli or in terms of a current task set or rule^38^. However, there are situations in which current stimuli do not in themselves provide the evidence necessary to make a choice. Whether the choice refers to the action itself, how to select it, when or whether to make it^52,53^ the resolution of ambiguity is time consuming and effortful, with extensive activations observed for what might otherwise be seen as trivial or inconsequential choices^27^. In neuroeconomic terms, there is a cost of ambiguity, to resolve a choice when the expected rewards are too similar between response options^54^.

One solution to the problem of ambiguity is stochastic decision-making, competing ‘first past the post’ between response options^11,44^. An arguably simpler strategy is to assign differential value according to a local arbitrary rule, thereby replacing the ambiguity by a value based decision process^55^, even without attributing stable or causal relationships between decision and outcome^56^. The presence of an arbitrary ‘rule’ would also reduce the differential reaction time between trial types, and support the observed attribution of value to freely chosen responses^57^.

As a result, a participant would have *a-priori* response preferences that determine the inequality in the distribution of ostensibly equivalent choices, despite the experimental neutrality over the response options. Such an arbitrary ‘rule’ would also reduce the differential reaction time between trial types. Rules and response-sets can be chosen^53^, but more usually they are specified experimentally. Multiple studies show the prefrontal and frontopolar cortical representations of such rules^36,38,53^ and the impact of prefrontal lesions on rule-guided or goal-directed action. The specific rule need not be directly determined, and may change over time, but such rules would reduce the entropy of responses.

We observed the neurophysiological correlate of selection entropy at right frontal sensors *before* the action was made. Source localisation of these correlations revealed peak responses in the right supramarginal gyrus at −340 ms, and right frontopolar prefrontal cortex and left orbitofrontal cortex at −16 ms. The prefrontal cortex is known to be active when learning or retrieving rules and when implementing or switching rules^37,44^. We suggest that the application of a local rule, by prefrontal cortex, is embedded in the current sequence of trial types and choices, whether a simple rule (e.g. the avoidance of repetition) or a more complex statistical dependency between events. Premotor and supplementary motor areas may also show activation in respond to arbitrary rules and in volitional actions^22,58^ but these areas were not observed for selection entropy correlations. We also observed activity related to selection entropy *after* the response in left orbitofrontal cortex and right frontopolar prefrontal cortex. We suggest this activity might be updating a monitor of past responses, although it could represent the reinforcement of the transient response set^36,38^. The prefrontal cortical correlations with selection entropy increased with longer window lengths, which argues against a within-trial ‘surprise’ signal^11,59^. Further studies could directly assess whether this activity is monitoring past responses by testing whether the activity predicts if the participant switches or repeats the previous response on the next response. However, with only 12.2% probability of repetition in trials we did not have enough trials to do this.

We consider the importance of these results in regards to the broader issue of a predictive brain that is sensitive to odd and unexpected events (e.g. the mismatch response^2,60^), regularities in stimulus patterns and sequence learning^61^ and here we show that such sensitivity also extends to regularity in action choices. Changes in neural responses to regularities in sensory sequences are shown to correlate with changes in different neurological and psychiatric disorders^62^. Therefore, it is of importance to conduct further studies into whether monitoring regularity in action choices also change in different disorders. We interpreted our MEG negative correlations in terms of a monitor of statistical regularities in sensory and motor events. However, the representation of statistical regularities equates to forming beliefs, including implicitly the beliefs used to make predictions about sensory inputs in a hierarchical predictive model of our actions and the environment. Indeed, it has recently been shown that mechanisms for monitoring prior stimulus statistics are represented at the neural level in rodents^63^, demonstrating that such processes have significant evolutionary relevance.

The experimental modulation of trial entropy was analogous to earlier studies of audio-visual sequence entropy^12,64^. Our negative MEG correlations were concordant with evidence for activation-entropy associations for auditory and visual sequences^15,65^. The negative correlations between neural activity and trial entropy replicated those demonstrated by Zhang and Rowe (2015). However, their fMRI study could not establish whether the activity occurred before or after the response. Here we demonstrate that neural activations related to trial entropy were limited to the post-response period and therefore represent the monitoring of recent events rather than an action selection process.

In contrast to previous studies that used either short fixed event lengths or entire sequences^14,16,59,66^, we varied windows lengths from 25 to 50 trials, in line with the neural representations of information theoretic measures such as entropy^11,15,66^. This length is in keeping with the predictive value of events on remote future choices observed in non-human primate decisions^67^. We observed increased negative correlations between prefrontal regions and trial and selection entropy for the longer trial windows, suggesting that stronger frontal activity supports the updating or consolidation of response sets over multiple trials.

Additionally, in a post-hoc analysis we observed an interaction between monitoring of events and the selection of action. For some individuals, periods of irregular events (high trial entropy) were associated with more regularity of action selection (low selection entropy). This behavioural interaction was significantly related to individual differences in the strength of activity related to monitoring recent trials (Fig. 5). This provided a neurophysiological marker of individual differences in the degree to which recent behaviors and stimuli constrain subsequent voluntary actions. The importance of frontal brain regions for monitoring volitional decisions and influencing future decisions is clear from the behavioural consequences of damage or degeneration of the regions we identify in association with action selection. These may impair the self-initiation of actions, as a feature of apathy^68^, reduce a sense of agency for one’s own actions^24^, or lead to perseverative and stereotyped behaviours.

The implication of the negative relationship between TE and SE is that the more entropic previous events have been, the less entropic an individual’s arbitrary choices tend to be. This can be seen as an analogous observation to inhibition of return in saccadic choices. Therefore, if the constrained trials are already highly entropic, it is not necessary to introduce entropy in one’s selections to avoid returning time and again to the same arbitrary choice. If a goal of the nervous system in arbitrary choices is to diversely sample selections to gain information about the relative merit of seemingly equivalent choices and thus constrain future choices in the optimal manner^69,70^, it is more informative in determining the optimal choice at any one instant, to use the fidelity with which one has monitored previous sampling not previous unconstrained choices. This view is supported by our observation that individual differences in the strength of the relationship between past events and future choices could be accounted for by the strength of neural monitoring of past events (TE) but not by the strength of monitoring of past choices (SE).

There are potential limitations this study. First, participant’s attention may have varied across the length of the task. We tested correlations between reaction times and selection entropy, hypothesising that reaction times increase with reduced attention: there was not a significant correlation. Second, we analyzed the differences between trial window lengths using a full factorial model and corrected for non-sphericity because of the high correlations between regressors of the different window lengths that were nested within sequence. An alternative approach could use a separate regression model for each window length, followed by a disjunction test to estimate the temporal specific MEG activation associations. Both methods were used by Zhang and Rowe (2015), with similar results. However, the disjunction test would only show regions that correlate in one trial window and not the other, rather than directly testing the hypothesis that window length is itself a determinant of neural activity. Third, the selection entropy correlations were only observed using a more lenient whole-head statistical correction than trial entropy correlations. However, the location of selection entropy correlates were in agreement with anatomical priors based on the fMRI study of this task^11^ and analogous correlates of regularity in other tasks^15,16^. Fourth, our study was designed and powered to examine main effects of the association between activity and TE/SE but we acknowledge that power was limited for our post-hoc assessment of the neural correlations with individual differences: we were powered to detect large effects (similar studies using fMRI range from 12–16 participants^11,15,16,45^). Despite this, we have demonstrated a robust statistical relationship between the degree to which individuals avoid repetition in their selected actions and the strength with which they neurally monitor the entropy of past events (trial entropy, Fig. 5a). We did not demonstrate a relationship between individuals’ actions and the strength with which they monitored their previous choices (selection entropy, Fig. 5b).

In conclusion, we propose that when choosing between alternative response options, healthy adults make their decision in part based on a monitor of past events. Statistical regularities in the preceding actions are updated over successive trials, represented in prefrontal areas, and thereby influence subsequent choices between otherwise equivalent responses. We suggest this strategy reduces cognitive effort, and obviates a pause of ongoing behavior during decision-making under uncertainty^42^. We show that individual differences in sequential decisions relate to the strength of prefrontal monitoring of regularities in previous actions more than to the neural effort associated with the instantiation of such rules. Damage to these monitoring and selection processes may contribute to the stereotypies, inflexible predictions or chaotic behavioral patterns arising from frontal-lobe neurological disorders^71–74^.

## Methods

### Participants, data collection and preprocessing

Twenty healthy, right-handed adults participated in the study (10 females, mean age 26.0 ± 4.9 years, range 18–37). Two participants were excluded from further analysis due to error rates greater than three standard deviations from the group mean. Participants gave informed written consent. The study was approved by the Cambridge 2 Research Ethics Committee and the methods were carried out in accordance with the relevant guidelines and regulations. Participants had no history of psychiatric or neurological illness, and no previous experience of the task.

### Task

We measured statistical regularities over temporally extended stimulus and action sequences using a multi-choice action selection task that allows participants to choose between action responses without explicit or learned rewards or feedback. The task has been used to study action decisions in healthy individuals^11^, in ageing^75^, and in Parkinson’s disease^71^, with robust patterns of activation at group- and single-subject levels^27^. In brief, participants watched an image of a hand with empty circles above the fingers. In ‘specified’ trials, a single circle was filled, cueing the participant to press the corresponding finger on a manual button box. In ‘chosen’ trials all four circles were filled, directing the participant to make a choice to press any one of their four fingers (Fig. 1a). Participants were asked to make a “fresh choice, regardless of what they had done before”, as quickly as possible. There were no reward differences between action choices, no feedback, and no suggestion of rules for particular modes of response (such as to be ‘random’). Null trials appeared identical to a prolonged inter-stimulus interval, with no response required of participants (to keep the paradigm identical to that used in previous fMRI studies, in which the null trials facilitate modelling). Stimuli were displayed for 1 second with 2.5 second stimulus onset asynchrony. The task contained 320 specified trials, 320 choice trials and 320 null events, were pseudo-randomly intermixed. The trials were split into four ten-minute blocks with short breaks (30s) for the participant to rest. The task was presented using E-Prime® software (Psychology Software Tools Inc.).

### MEG data acquisition and processing

MEG data were collected using a magnetically shielded 306-channel Vectorview system (Elekta Neuromag), with a magnetometer and two orthogonal planar gradiometers at each of the 102 sensor positions. Vertical and horizontal eye movements were recorded using paired EOG electrodes, and the head position was monitored using five head-position indicator coils. A 3D digitizer (Fastrak; Polhemus) was used to record the three-dimensional locations of the coils, three anatomical fiducials (nasion and left and right preauricular points) and approximately 100 scalp points. We used Maxfilter software to make adjustments for head movement^76^, and to downsample the data from 1 kHz to 250 Hz.

The remaining pre-processing steps were completed using SPM12 software (Wellcome Department of Imaging Neuroscience, London, UK). We high-pass filtered at 0.1 Hz and low-pass filtered at 40 Hz using Butterworth filters. We epoched the data around the participant’s action response from −1500 ms to 1500 ms. We applied a baseline correction from −1500 to −1000 ms to ensure a baseline before the cue presentation. We used baseline correction because of our interest of how the evoked response is modulated by context of SE and TE, rather than the mean effect of these entropy measures over time. We applied automatic trial artefact rejection by thresholding the EOG electrodes at 200 µV. Omission and commission error trials were rejected, as were trials on which the participant’s reaction time was less than 150 ms or longer than 1500 ms.

### Sliding window entropy measures of randomness

To investigate neural monitoring of temporal events, we calculated the entropy measures of previous trials and of previous action selection events, replicating Zhang and Rowe (2015). We correlated these entropy measures with single-trial MEG responses. We calculated the entropy over a sliding window of previous stimuli (*C*_*j*_ = {*a, b, c, d, e*}, Fig. 1b) or previous choice selection responses (*A*_*j*_ = {*1, 2, 3, 4*}, for each finger). Iterating through each trial *i*, the window included the range of trials: [*i-n+1, i*], where *n* was the length of the window (Fig. 1c). The entropy measure was then assigned to the *i*^*th*^ trial and correlated with that trial’s MEG response. Though error trials were excluded from imaging statistics, they were included in the measurement of trial entropy (TE) to obtain the complete measure of stimulus trial variability. Choice trial omission errors were not included in the calculation of selection entropy (SE). The entropy values were calculated for six trial windows (*n* = 25–50 trails, step size = 5), to examine whether any brain regions were sensitive to the fluctuations of entropy over different timescales^11^.

TE was defined by Shannon’s entropy. TE quantitatively measures the degree of randomness in the presented stimuli within the sliding window. Higher TE values indicated higher randomness within the window. The TE at the *i*^*th*^ trial is given by:

1 $$TE(i)=H(Stimuli)=-\,\sum _{k=(a,b,c,d,e)}p({C}_{j}=k)\mathrm{log}\,p({C}_{j}=k),(i-n+1\le j\le i)$$

SE was measured from the degree of randomness in participants’ responses in the choice trials. SE was defined by conditional entropy^15^, calculating the probability of each action given the stimuli was a choice trial. The SE at the *i*^*th*^ trial is given by:

2 $$\begin{array}{rcl}SE(i) & = & H(Actions|Stimuli=\{e\})\\  & = & -\,\sum _{\begin{array}{c}k=(e)\\ m=\{1,2,3,4\}\end{array}}p({A}_{j}=m,{C}_{j}=k)\mathrm{log}\,p({A}_{j}=m|{C}_{j}=k),\end{array}\,(i-n+1)\le j\le i)$$

TE and SE values were not calculated for the trials occurring before the end of the first sliding window of interest because there were not enough trials to calculate the entropy measure. Both TE and SE were calculated for every following trial, whether specified or choice, because we assumed each measure to be a sustained state representation of the degree of order based on recent trials^11^.

### Entropy-related MEG responses

For each participant, their trial-by-trial TE and SE measures were mean centred and used as regressors within a first-level general linear model to correlate with the single-trial planar-gradiometer MEG data. We firstly completed our analyses in MEG sensor space to be able to observe the correlations across all space and all time. This analysis was repeated for each sliding window. For both TE and SE, we included the first-level contrast images across all six windows within a second-level full-factorial model to examine neural correlations of entropy for all trial window lengths and to contrast these correlations across window lengths. The data were adjusted for unequal variance and for non-sphericity with dependence between measures. Note that a repeated measures design was not appropriate for the multiple window lengths, as the measures reflected nested sequences and were not independent repeated samples. We analyzed trials locked to the participants’ responses to investigate temporal precedence of these neural correlations in relation to the action response.

### Source Localisation of Entropy correlations

Additionally, we performed single-trial MEG source localisation to make inferences of the neural sources that correlated with the measures of entropy. Firstly, we estimated the forward leadfield model using the participant’s individual structural MRI scan to construct a realistic single-shell head model, normalised to MNI standard space (MRI: 3T Siemens Tim Trio, T1-weighted 3D MPRAGE sequence, TR = 2250 ms, TE = 2.99 ms, flip angle 9°, field-of-view 240 × 256 × 160, 1 mm slice thickness). The head model was co-registered to digitised anatomical fiducial markers and scalp points. We computed the inverse source reconstruction for every trial using the minimum norm algorithm^77^ for 20 ms time windows around the time of peak significance for each of the significant clusters observed in the sensor space analysis. The resulting single-trail source reconstructed images were smoothed using an 8 mm FWHM Gaussian kernel and then correlated with the trial and selection entropy values, replicating the statistical analysis steps used in the sensor space correlations. As the goal of the reconstructions was to visualise the location of the neural sources already statistically demonstrated in sensor space, correlation maps were displayed at a voxelwise threshold of p < 0.01.

### Inter individual variability

TE and SE are negatively correlated^11^. We observed that the strength of this relationship differs between individuals (Fig. 2d). To assess whether this coupling between TE and SE related to the strength of entropy-related neural activity, we undertook a further three step post-hoc analysis. First, we separately extracted binary masks of the scalp topology of neural activity related to TE and SE from the general linear model described above (Figs 3a and 4a). Second, using these masks as a region of interest, for each individual we separately extracted the peak t-score between neural coupling and both TE and SE, reflecting the strength of the neural response associated with these measures. T-scores are a more appropriate measure here than beta estimates, as they are less vulnerable to differences in noise between scalp locations at the single pixel level^78^, and in overall response amplitude between brain areas^79^. Finally, we correlated these extracted values, which represented the individual strengths of coupling between neural activity and either TE or SE, with subjects’ behavior calculated as follows: to account for the possibility of a delayed relationship between TE and SE, for each individual we aligned the TE and SE signals by maximising the normalized cross correlation within a lag window of ±10 trials. At this optimal lag, we then calculated the Pearson’s product moment correlation co-efficient between TE and SE at the window length of 25 trials. This window length was chosen because, as shown in Fig. 2c, the absolute value of the TE-SE correlation was relatively unaffected by increasing window length but the variability of the measure increased. As can be appreciated from Fig. 2a and b, at longer window lengths much of the trial-to-trial variability in TE and SE is reduced by temporal smoothing. A short window length therefore allows the most robust measure of TE-SE correlation. These data were then Fisher Z-transformed for correlation with the measure of neural activity.

### Data availability

The data that support the findings of this study are available from the corresponding author upon reasonable request, for academic (non-commercial) purposes.
